# Inactive lifestyles and sedentary behavior in persons with chronic aneurysmal subarachnoid hemorrhage: evidence from accelerometer-based activity monitoring

**DOI:** 10.1186/s12984-017-0331-1

**Published:** 2017-11-23

**Authors:** Wouter J. Harmsen, Gerard M. Ribbers, Majanka H. Heijenbrok-Kal, Johannes B. J. Bussmann, Emiel M. Sneekes, Ladbon Khajeh, Fop van Kooten, Sebastian J. C. M. M. Neggers, Rita J. van den Berg-Emons

**Affiliations:** 1 Rijndam Rehabilitation Institute, Rotterdam, the Netherlands; 2 Department of Rehabilitation Medicine, Erasmus MC University Medical Center Rotterdam, P.O. Box 2040, 3000 CA Rotterdam, The Netherlands; 3 Department of Neurology, Erasmus MC University Medical Center Rotterdam, Rotterdam, the Netherlands; 4 Department of Endocrinology, Erasmus MC University Medical Center Rotterdam, Rotterdam, the Netherlands

**Keywords:** Subarachnoid hemorrhage, Physical activity, Sedentary behavior, Fatigue, Rehabilitation, Stroke

## Abstract

**Background:**

Aneurysmal subarachnoid hemorrhage (a-SAH) is a potential life-threatening stroke. Because survivors may be at increased risk for inactive and sedentary lifestyles, this study evaluates physical activity (PA) and sedentary behavior (SB) in the chronic phase after a-SAH.

**Methods:**

PA and SB were objectively measured at six months post a-SAH with an accelerometer-based activity monitor, with the aim to cover three consecutive weekdays. Total time spent in PA (comprising walking, cycling, running and non-cyclic movement) and SB (comprising sitting and lying) was determined. Also, in-depth analyses were performed to determine the accumulation and distribution of PA and SB throughout the day. Binary time series were created to determine the mean bout length and the fragmentation index. Measures of PA and SB in persons with a-SAH were compared to those in sex- and age-matched healthy controls.

**Results:**

The 51 participants comprised 33 persons with a-SAH and 18 controls. None of the participants had signs of paresis or spasticity. Persons with a-SAH spent 105 min/24 h being physically active, which was 35 min/24 h less than healthy controls (*p* = 0.005). For PA, compared with healthy controls, the mean bout length was shorter in those with a-SAH (12.0 vs. 13.5 s, *p* = 0.006) and the fragmentation index was higher (0.053 vs. 0.041, *p* < 0.001). Total sedentary time during waking hours showed no significant difference between groups (514 min vs. 474 min, *p* = 0.291). For SB, the mean bout length was longer in persons with a-SAH (122.3 vs. 80.5 s, *p* = 0.024), whereas there was no difference in fragmentation index between groups (0.0032 vs 0.0036, *p* = 0.396).

**Conclusions:**

Persons with a-SAH are less physically active, they break PA time into shorter periods, and SB periods last longer compared to healthy controls. Since inactive lifestyles and prolonged uninterrupted periods of SB are independent risk factors for poor cardiovascular health, interventions seem necessary and should target both PA and SB.

**Study registration:**

Dutch registry number: NTR 2085.

## Background

Aneurysmal subarachnoid hemorrhage (a-SAH) is defined by the extravasation of blood into the subarachnoid space, and is caused by a spontaneous bleeding of a ruptured brain aneurysm [1]. It accounts for 5% of all stroke cases and has an incidence rate of 9/100,000 persons/year and a mortality rate of 50% [1, 2]. Persons with a-SAH are relatively young compared with those with ischemic or hemorrhagic stroke (55 years vs. 70 years) [1, 3]. Further, whereas ischemic or hemorrhagic stroke may lead to focal brain injury with specific stroke-related symptoms, brain injury in a-SAH has a more diffuse character without typical stroke symptoms [1]. Those who survive are likely to experience long-term symptoms, such as cognitive problems (40%), emotional complaints (50%), depressive symptoms (40%), and fatigue (up to 90%) [4–7]. Even among those who are classified as having a ‘favorable outcome’, the incidence of clinical deficits is high [8].

Persons with a-SAH seem to have difficulty with resuming their daily activities, and only one-third is able to fully resume their previous occupation [4, 9]. The inability to perform daily activities is associated with passive coping styles, depressive symptoms and fatigue [6, 8–10]. Reduced physical fitness after a-SAH has been reported, [11]. which may also hinder the performance of daily activities. Therefore, individuals with a-SAH may be at risk of inactive and sedentary lifestyles, placing them at risk for poor health outcomes [12–14]. However, measures of daily PA and SB have not yet been studied after a-SAH.

PA refers to *‘any bodily movement produced by skeletal muscles that requires energy expenditure’* and contributes to the primary and secondary prevention of chronic diseases, including cardiovascular disease, cancer, diabetes mellitus, hypertension and obesity [15, 16]. SB, defined as a distinct class of activities that requires low levels of energy expenditure and involves sitting and lying activities during waking hours, negatively impacts metabolism and cardiovascular health [17, 18]. Recent studies show that SB impacts cardiovascular health, independent of the volume of PA [18]. Furthermore, not only the total volume of PA or SB, but also the way PA and SB are accumulated is important, i.e. prolonged bouts of PA are beneficial, whereas prolonged bouts of SB are found to be detrimental to cardiovascular health [18–21].

Persons with stroke not caused by a-SAH are highly sedentary, with PA levels almost half that of healthy control subjects [22–24]. In stroke rehabilitation, improving PA and SB is strongly recommended, as it provides protective benefits in the primary and secondary prevention of chronic diseases [16, 25]. Inactive and sedentary lifestyles in ischemic or hemorrhagic stroke have been frequently explained by motor impairments following neuro-motor lesions. Since brain injury in a-SAH is more diffuse without typical stroke symptoms (such as paresis), it would be of interest to gain insight in the level of PA and SB in this patient group.

Despite its importance, PA and SB have not yet been studied in persons with a-SAH. Therefore, this study evaluates PA and SB in the chronic phase after a-SAH. Objectively obtained measures of PA and SB were compared to those in sex- and age-matched healthy controls. This study can help to optimize recommendations to prevent chronic diseases and debilitating conditions after a-SAH, but can also be used to better understand the consequences of different types of stroke on daily PA and SB. Since individuals with a-SAH have difficulty in resuming their daily activities and have reduced physical fitness, we hypothesized that they would be less physically active and more sedentary compared to healthy controls.

## Methods

### Participants and study design

The present study (entitled HIPS-Rehab) was part of the ‘Hypopituitarism In Patients after Subarachnoid hemorrhage (HIPS) study’ [26]. In this study we investigate the PA and SB of persons who were six months post a-SAH. Participants with a-SAH admitted to the department of Neurology of Erasmus University MC were eligible for inclusion if they were aged ≥18 years. Diagnosis of a-SAH was confirmed by computerized tomography (CT) of the brain and, in cases with negative CT, by lumbar puncture. Exclusion criteria were: hypothalamic or pituitary disease diagnosed prior to a-SAH, history of cranial irradiation, trauma capitis prior to a-SAH, other intracranial lesion apart from a-SAH, and other medical or psychiatric condition or laboratory abnormality that may interfere with the outcome of the study. Participants were also excluded if they were aged ≥70 years. For comparison, we included healthy controls of similar sex (% females; 64 vs. 72%, *p* = 0.382) and age (52.6 vs. 51.0 years, *p* = 0.548). Healthy controls were recruited by advertisement; they wore identical activity monitors and similar measurement procedures were applied.

The study was approved by the Medical Ethics Committee of Erasmus University Medical Centre, and all participants provided written informed consent.

### Physical activity and sedentary behavior

**Fig. 1:**
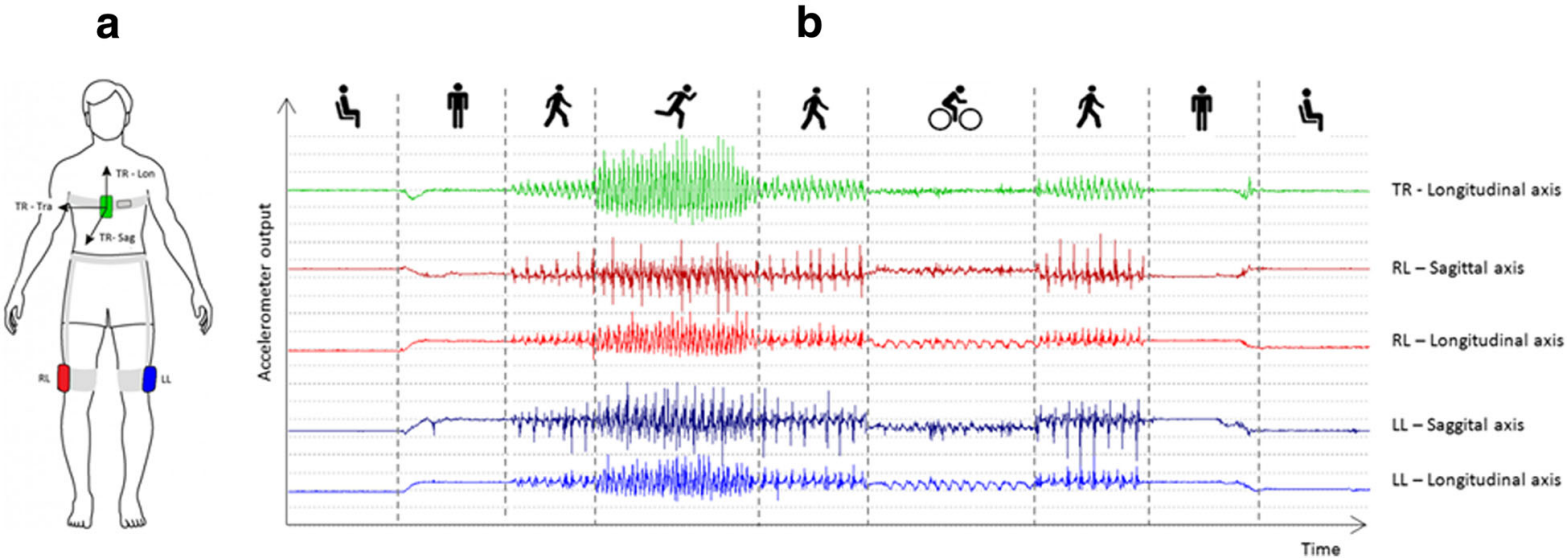
Placement of the VitaMove activity monitor is shown in (**a**), and a typical output derived from the raw accelerometer signals is presented in (**b**). The pattern of these signals determines the corresponding activity. A sequence of activities is presented: sitting, standing, walking, running, walking, cycling, walking, standing and sitting. Abbreviations: TR = Trunk sensor; RL = Right leg sensor; LL = Left leg sensor; Lon = longitudinal axis; Sag = sagittal axis; Tra = transversal axis

PA and SB were objectively measured with an accelerometer-based activity monitor (VitaMove)^1^ (Fig. 1). This monitor has demonstrated validity for quantifying body postures and movements in healthy subjects and in different patient groups [27–29]. The VitaMove activity monitor consists of three individual body-fixed recorder units, which are wirelessly connected and synchronized every 10 s. One recorder unit was attached to the trunk (sternum position) and one to each thigh, using specially developed elastic belts. Each unit has its own tri-axial accelerometer,^2^ power supply and storage capacity. Participants wore the VitaMove on consecutive weekdays, except during swimming, bathing and sleeping. In line with previous research, the intended duration of measurement was three consecutive days, with a minimum of one day [30, 31]. Further, the signal processing parameters were identical to the parameter settings used in previous validity studies [27–29]. Mean values were calculated for multiple days of activity monitoring. Participants were instructed to continue their ordinary daily activities. The principles of the measurements were explained after all measurements were completed in order to avoid measurement bias. In addition, participants kept activity diaries to report reasons of non-wear periods of the activity monitor.

### Data processing

Accelerometer signals of each recorder unit were continuously measured and stored (128 Hz) on a micro Secure Digital memory card. Accelerometer signals were downloaded on a computer for kinematic analyses using specially developed VitaScore software.^3^ Waking hours were determined by the researcher (WH) using the diaries filled out by the participants and by inspection of the raw data signals; specifically, ongoing flatlines indicate that the recorder units were taken off, reflecting the end of waking hours. Body postures and movements (e.g. lying, sitting, standing, walking, cycling, running and non-cyclic movements) were automatically detected with a 1-s time resolution from the feature time series (i.e. angle, frequency and motility) derived from the measured accelerometer signals. The motility feature expresses the intensity of the movement of the body segment to which the unit is attached, and depends on the variability of the acceleration signal; motility can be compared to counts that are calculated in regular activity monitors (calculated in gravitational force (g), 1 g = 9.81 m/s^2^). During walking, the body motility signal (i.e. the mean of the leg and trunk motility signals) corresponds to walking speed [32]. The minimum duration threshold for each activity was 5 s. A detailed description of the algorithms and analysis is published elsewhere [28, 30].

In-depth analyses were performed to quantify the accumulation and distribution metrics of PA and SB. For PA outcomes, the four detected body movements (walking, cycling, running, and non-cyclic movements) were categorized into one PA category; a similar procedure was followed for the SB category, covering lying and sitting activities. Binary time series of PA (yes = 1, no = 0) and SB (yes = 1, no = 0) were created using custom-made Matlab algorithms. A period of uninterrupted samples of either PA (or SB) was classified as a bout. Due to the minimum duration threshold of 5 s, bouts and periods between bouts lasted at least 5 s.

### Outcome measures

#### Volume, intensity and distribution of PA and SB

To determine the volume of PA, we calculated the total time spent in the four detected body movements during waking hours. The volume of total SB was determined by evaluating the total time of sitting and lying activities during the waking hours. Volume measures were then expressed as a percentage of a 24h period, and as a percentage of waking hours. The mean motility of PA and the mean motility of walking were also determined and expressed in g.

The binary time series were used to determine the accumulation and distribution of PA and SB. The total number of bouts and the mean bout length (in seconds) were calculated. Since the mean bout length was not normally distributed, the natural logarithm was taken. The mean log length was back transformed to the original scale. The fragmentation index was calculated and reflects the ratio between the number of PA (or SB) bouts divided by the total PA (or SB) time [33]. A higher fragmentation index indicates that total PA (or SB) time is more fragmented, which means that there are less prolonged periods of PA (or SB) [34].

#### Participants’ characteristics

At hospital intake, the following Clinical characteristics were obtained including: 1) the severity of a-SAH according to the grading of the World Federation of Neurologic Surgeons (low-grade: I-III or high-grade: IV-V) [35, 36] and the Glasgow Coma Scale (GCS) score, [37] 2) location of the aneurysm (anterior or posterior circulation), 3) treatment procedure (surgical clipping or endovascular treatment), 4) presence of secondary health complications (re-bleed, delayed cerebral ischemia, hyponatremia, hydrocephalus and growth hormone deficiency; defined as an insufficient growth hormone (GH) response to a GH-releasing hormone -arginine test), [38] and 5) neurologic comorbidity (paresis or spasticity). Neurologic morbidity (such as paresis or spasticity) was evaluated by treating neurologist. Information on the following characteristics and body anthropometrics were collected from both groups: sex, age, weight, height and Body Mass Index (BMI).

## Statistical analysis

All data are expressed as mean (SD) unless otherwise indicated. To compare the clinical characteristics between participants of HIPS-Rehab and those who did not participate (but were included in HIPS), we used independent t-tests for continuous data and chi-square-tests for categorical data. To compare the characteristics and measures of PA and SB between individuals with a-SAH and controls, independent t-tests were applied for continuous data and chi-square tests for categorical data. All analyses were performed using IBM SPSS Statistics, version 20.^4^ A probability value of *p* < 0.05 was considered statistically significant.

## Results

**Fig. 2:**
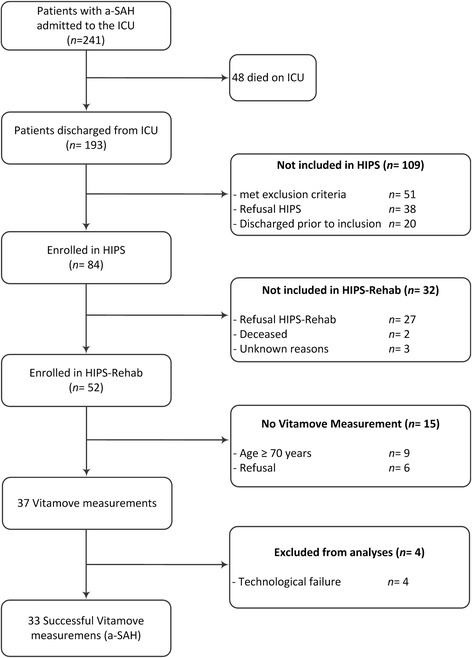
Consort flow diagram

Of the 241 patients admitted to the ICU with a diagnosis of a-SAH, 84 were included in HIPS of which 52 volunteered to participate in HIPS-Rehab. Participants in HIPS-Rehab (*n* = 52) did not differ from those who did not participate (but were included in HIPS; *n* = 32) regarding the severity of a-SAH, location of the aneurysm, treatment procedure, and the presence of secondary health complications. Of the 52 participants, successful activity monitoring measurements were obtained from 33: of the 19 unsuccessful attempts, 6 refused to wear the activity monitor, in 4 persons data processing was unsuccessful due to technological failure, and 9 were aged ≥70 years (Fig. 2).

Table 1 presents the clinical characteristics. Most persons underwent endovascular coiling (82%) and had a ruptured aneurysm in the anterior circulation (61%). The neurological scores showed that 29 participants had a low-grade a-SAH (88%) and a mean GCS score of 14.0 (SD 2.0). None of the participants had a paresis or showed signs of spasticity.

**Table 1:** Descriptive characteristics

Characteristics	Participants with a-SAH (*n* = 33)
Sex, female, *n* (%)	21 (64%)
Age (years), *mean (SD)*	52.6 (9.0)
*WFNS grade, n* (%)	
I	16 (48%)
II	13 (39%)
III	1 (3%)
IV	3 (9%)
V	0
Glasgow Coma Scale score, *mean (SD)*	14.0 (2.0)
Location aneurysm, *n* (%)	
Anterior circulation	20 (61%)
Posterior circulation	13 (39%)
Aneurysm treatment, *n* (%)	
Endovascular treatment^a^	27 (82%)
Surgical clipping	6 (18%)
Re-bleed, *n* (%)	0
Delayed cerebral ischemia, *n* (%)	7 (21%)
Hyponatremia, *n* (%)	4 (12%)
Hydrocephalus, *n* (%)	9 (27%)
Growth Hormone Deficient, *n* (%)	2 (6%)

*Abbreviations: WFNS grade* World Federation of Neurologic Surgeons grading system for subarachnoid hemorrhage

^a^Endovascular treatment is also known as endovascular coiling

Due to challenges with activity monitoring, data were not available for all participants for the intended three days of measurement. The duration of measurement was 3 days in 42% of those with a-SAH and in 83% of the controls; 2 days in 48% of those with a-SAH and in 6% of the controls; and 1 day in 9% of the persons with a-SAH and in 11% of the controls. Mean daily wear time did not differ between the groups, 13.7 h (SD 1.8) vs. 14.1 h (SD 1.3), respectively, (95% CI of the difference: −1.4 h to 0.5 h; *p* = 0.372).

Table 2 presents the characteristics of the two groups: persons with a-SAH did not differ from healthy controls regarding sex (*p* = 0.382), age (*p* = 0.548), weight (*p* = 0.231) and height (*p* = 0.062), but had a higher BMI (*p* = 0.002). Table 2 also presents the volume measures of PA and SB in the two groups. Persons with a-SAH spent 105 min/24 h (=7.3%) being physically active, which is 35 min/24 h (2.4%) less compared with that of healthy controls (140 min/24 h (=9.7%); *p* = 0.005); in particular, there was less participation in cycling activities (3 min/24 h (=0.2%) vs. 27 min/24 h (=1.9%); *p* < 0.001). Total sedentary time did not differ between those with a-SAH and healthy controls; 514 min/24 h (=35.7%) vs. 473 min/24 h (=32.9%; *p* = 0.291), respectively. Also, there was no difference between the groups in total standing time, i.e. 200 min/24 h (=13.9%) vs. 233 min/24 h (=16.2%, *p* = 0.164), or in mean PA motility and mean walking motility (*p* = 0.442 and *p* = 0.503, respectively).

**Table 2:** Characteristics and results of activity monitoring measurement

	Participants with a-SAH (*n* = 33)	Healthy controls (*n* = 18)	95% CI of the difference	*p-value*
Sex, females, *n* (%)	21 (64%)	13 (72%)		0.382
Age (years)	52.6 (9.0)	51.0 (8.6)	−3.7 to 6.8	0.548
Weight (kg)	75.6 (13.0)	71.4 (9.1)	−2.7 to 11.1	0.231
Height (m)	1.67 (0.1)	1.72 (0.1)	−11.7 to 0.3	0.062
Body Mass Index (kg/m^2^)	27.1 (3.8)	23.9 (2.0)	1.2 to 4.7	0.002
Activity profiles^a^				
% Physical activity	7.3 (3.0)	9.7 (2.1)	−3.9 to − 0.8	0.005
- % Walking	4.7 (2.4)	4.7 (1.3)	−1.2 to 1.3	0.964
- % Cycling	0.2 (0.4)	1.9 (1.7)	−2.3 to − 1.1	<0.001
- % Running	0.0 (0.0)	0.1 (0.3)	−0.2 to − 0.02	0.018
- % Non-cyclic movement	2.4 (1.7)	3.0 (1.1)	−1.5 to 0.3	0.200
% Standing	13.9 (5.4)	16.2 (5.7)	−5.6 to 1.0	0.164
% Sedentary behavior	35.7 (9.9)	32.9 (7.6)	−2.5 to 8.3	0.291
- % Sitting	32.5 (10.1)	30.7 (7.7)	−3.6 to 7.3	0.502
- % Lying	3.2 (3.5)	2.2 (1.8)	−0.8 to 2.8	0.253
% Non-wear^b^	43.0 (7.3)	41.2 (5.6)	−2.2 to 5.8	0.372
Intensity meausures				
Motility physical activity (g)^c^	45.6 (11.9)	47.8 (8.6)	−7.5 to 3.3	0.442
Motility walking (g)^c^	37.6 (9.9)	39.7 (7.7)	−8.6 to 4.3	0.503

Duration of activities as a percentage of 24 h

^a^Physical behavior was monitored on consecutive weekdays in the free-living situation

^b^Non-wear = time that participants did not wear the activity monitor (also reflects nighttime)

^c^g = gravitational forces *100

Mean bout length of PA in persons with a-SAH was shorter than in controls (12.0 s vs. 13.5 s; *p* = 0.006), and the PA fragmentation index was higher (0.053 vs. 0.041; *p* < 0.001), indicating that the PA periods were shorter and the total time spent in PA was more fragmented in persons with a-SAH. Mean bout length of SB was longer in persons with a-SAH (122.3 s vs. 80.5 s; *p* = 0.024), whereas the SB fragmentation index did not differ between groups (*p* = 0.396). This indicates that SB periods lasted longer in persons with a-SAH, but the way in which the total SB was distributed did not differ between groups (Table 3).

**Table 3:** Distribution of physical activity and sedentary behavior

	Participants with a-SAH (*n* = 33)	Controls (*n* = 18)	95% CI of the difference	*p-value*
Physical activity				
Mean bout length (s)^a^	12.0 (1.9)	13.5 (1.2)	−2.4 to −0.4	0.006
Fragmentation index^b^	0.053 (0.01)	0.041 (0.01)	0.006 to 0.019	<0.001
Sedentary Behavior				
Mean bout length (s)^a^	122.3 (71.5)	80.5 (35.6)	5.6 to 78.0	0.024
Fragmentation index^b^	0.0032 (0.002)	0.0036 (0.002)	−0.001 to 0.001	0.396

The minimum bout length lasted at least 5 s

^a^Uninterrupted bouts with a minimum length of 5 s

^b^Fragmentation index represents the ratio between the number of sedentary bouts divided by the total time being sedentary

## Discussion

The present study shows that persons with a-SAH are less physically active, they break PA time into shorter periods, and SB periods last longer compared to healthy controls, placing them at increased risk for poor health outcomes [12–14]. This is the first study on PA and SB in persons with a-SAH. The objectively obtained measures of PA and SB have meaningful implications for stroke rehabilitation because our findings reveal that inactive and sedentary lifestyles are present in absence of motor impairments. Given the importance of optimal PA and SB, [21, 22] therapeutic interventions are warranted. The present findings may help to improve interventions (targeting both PA and SB) and prevent debilitating conditions after a-SAH.

In-depth analysis of PA revealed that persons with a-SAH break their PA time into shorter periods, which is not beneficial from a health perspective [21]. These interruptions may be explained by an increased number of moments of rest, possibly related to higher fatigability, cognitive dysfunction, and/or lower cardiorespiratory fitness [5, 7]. The most recent guidelines of the WHO recommend an accumulation of PA time, i.e. uninterrupted PA, of at least 10 min, as this is an important aspect of healthy PA [39]. Therefore, therapeutic interventions should not only target the total volume of PA, but should also improvethe accumulation of PA time in persons with a-SAH.

Sedentary time, particularly accumulated in long uninterrupted periods, negatively impact cardiovascular health, independent of the volume of PA [18]. In-depth analysis of SB revealed that SB periods lasted longer in persons with a-SAH. However, the SB fragmentation index did not differ, indicating that the way total SB is distributed in a-SAH is similar to that in healthy controls. This could be explained by the fact that the total sedentary time was somewhat higher (albeit not significant) in a-SAH than in healthy controls. Since SB periods lasted longer in those with a-SAH than in controls, breaking prolonged uninterrupted SB periods may represent another therapeutic target in order to provide additional health benefits in persons with a-SAH.

In patients with stroke not caused by a-SAH, the most commonly used objective measures of PA are step or activity counts per day; these counts are reported to be almost half those of healthy controls [22–24]. The present study explored activity profiles beyond simple step or activity counts and distinguished different types of PA. Overall, persons with a-SAH spent 25% less time in PA than healthy controls (105 vs 140 min/24 h, respectively). However, the total volume of walking activities did not differ between groups; this is in line with an accelerometer-based study on walking activities in patients with stroke [40]. Furthermore, compared with controls, persons with a-SAH participated particularly less in cycling activities and, to a lesser extent, in running activities.

Interestingly, physically inactive and sedentary lifestyles after a-SAH seem not be related to motor impairments, and therefore other mechanisms should underlie our findings. For example, PA may be limited by impaired cardiorespiratory fitness, cognitive dysfunction, anxiety or fatigue. Feelings of anxiety after a-SAH can highly restrict participation in daily activities [41, 42]. Furthermore, PA can also be limited by concentration problems, [5, 43, 44] e.g. cycling activities are more demanding due to participation in traffic and multitasking. However, future studies are warranted to investigate the barriers and facilitators of PA after a-SAH, and should take into account mechanisms of physical deconditioning, cognitive dysfunction, anxiety and fatigue.

In persons with a-SAH, total sedentary time during waking hours was 514 min. This is similar to findings in persons with stroke, not caused by a-SAH (i.e. sedentary times ranging from 464 to 654 min) [23, 24]. This is remarkable because patients with a *non* a-SAH stroke are often older and more restricted in the performance of daily activities (often because of neurological deficits) than patients with a-SAH [1, 6]. With regard to SB, there are no guidelines for the general population. A meta-analysis showed that above 7.0 h, every additional hour increase in SB time is associated with a 5% increase in all-cause mortality [45]. In the present study, individuals with a-SAH spent about 8.5 h being sedentary, implying a 7.5% increase in all-cause mortality. In order to set therapeutic targets, additional studies are needed to establish guidelines for SB.

The major strength of the present study is the objective measurement of PA and SB, without possible bias from the subjective character of a questionnaire. Also, the inclusion of healthy controls allowed us to better interpret the data. Another strength is that we used innovative in-depth analyses of PA and SB which provides new insights to support future therapeutic interventions.

### Study limitations

Some limitations of the present study should be discussed. First, we used an advanced activity monitor that allowed to obtain continuous data on various types of PA and SB. However, this makes it difficult to compare our data with general guidelines for healthy PA or SB, because these guidelines are mostly based on self-report questionnaires [29]. Future studies need to define guidelines for healthy PA and SB, based on objectively obtained measures. Second, for logistic reasons, the sample size of healthy controls was smaller compared to that in persons with a-SAH. Smaller number of controls have been frequently reported in activity monitoring research across different patient groups, including stroke [46, 47]. Overall, results on the main outcome (volume metrics) in the controls are comparable with, and for PA even somewhat lower (9.7% vs 10–12% per 24 h, respectively) than results, as measured with the VitaMove, in other healthy comparison groups [48–50]. This difference may even indicate that we have underestimated the lack of PA in persons with a-SAH. BMI was somewhat higher in persons with a-SAH than in controls. However, it was not feasible to account for BMI, as a higher BMI may already be indicative of physically inactive and sedentary lifestyles. Another limitation is that, in both groups, actually ‘wearing’ the activity monitor may have influenced PA in daily life; nevertheless, all participants reported that they performed their regular PA. Another limitation is that we did not include any physiological parameters (e.g. heart rate) that might have provided more details on physical strain of PA in daily life.

## Conclusions

Objectively obtained measures of PA and SB show that persons with a-SAH are less physically active, they break PA time into shorter periods, and SB periods last longer compared to healthy controls. These results suggest that persons with a-SAH have increased health risks related to inactive lifestyles and sedentary lifestyles. Given the importance of optimal PA and SB, future studies need to identify barriers and facilitators of PA and SB to develop optimal therapeutic interventions to improve PA and SB after a-SAH.

## Footnotes

1 Activity Monitor, VitaMove, 2 M Engineering, Veldhoven, the Netherlands

2 Accelerometer, Freescale MMA7260Q, Denver, USA

3 Activity monitor software, VitaScore BV, Gemert, the Netherlands

4 SPSS Inc., Chicago, IL, USA
